# *Botanophila* flies, vectors of *Epichloë* fungal spores, are infected by *Wolbachia*

**DOI:** 10.1080/21501203.2018.1515119

**Published:** 2018-09-08

**Authors:** Lydia Pagel, Thomas Bultman, Karolina Górzyńska, Marlena Lembicz, Adrian Leuchtmann, Anne Sangliana, Nicola Richards

**Affiliations:** a Biology Department, Hope College, Holland, MI, USA; b Department of Plant Taxonomy, A. Mickiewicz University, Poznan, Poland; c Institute of Integrative Biology, ETH Zurich, Zurich, Switzerland; d Biocontrol & Biosecurity, AgResearch, Lincoln Science Centre, Lincoln, Canterbury, New Zealand

**Keywords:** Ascomycota, Diptera, fungal endophyte, sexual parasite

## Abstract

*Epichloë* fungi are endophytes within grasses that can form stromata on culms of their hosts. *Botanophila* flies visit the stromata for egg laying and in the process can vector spermatial spores, thereby cross fertilising the fungus. Following egg hatch, larval flies consume fungal tissue and spores. Thus, *Epichloë* individuals with traits that limit larval consumption could be at a selective advantage. We assessed *Botanophila* fly larvae from sites within the United States and Europe for infection by the bacterial sexual parasite *Wolbachia* through amplification of the *Wolbachia* surface protein gene (*wsp*). Nearly 70% of fly larvae in our samples were infected by *Wolbachia*. This is the first record of infection by *Wolbachia* within *Botanophila* and could have far reaching effects on not only the fly host, but also the *Epichloë* fungi upon which *Botanophila* feeds as well as the grass host within which the fungi live. For example, infection by *Wolbachia* could limit consumption of *Epichloë* spores by *Botanophila* larvae if the bacteria promoted premature larval death.

## Introduction

The relationship between *Botanophila* flies and *Epichloë* fungi has long been a subject of interest to researchers due to the fly’s peculiar pollinator-like behaviour. This quasi-pollination interaction closely resembles that observed between some insects and their angiosperm hosts, such as the fig-fig wasp and yucca-yucca moth interaction, but is uncommon among fungi (Bultman 1995). Flies find stromata by tracking volatiles the fungi produce (Steinebrunner et al. 2008a.). Once they locate a stroma, they alight and feed on perithecial tissues containing spermatial spores, and then oviposit and defecate along the whole length of the stroma. Spermatia pass through the gut of the fly unharmed and are deposited on subsequent stromata the fly visits. *Epichloë* fungi are self-incompatible and thus, flies cross-fertilise fungi as they vector spermatia (Bultman et al. 1998). Fly larvae remain on fungal stromata until just before pupation and feed on the products of cross fertilisation; the ascospores (Bultman et al. 1995). *Botanophila* flies appear to be the main vectors of spores although other vectors, like slugs, have been implicated (Rao et al. 2012; Hoffman and Rao 2014).

An enigma regarding mutualisms is their observed stability (Bronstein 2001, 2009). What prevents one party from over-exploiting the other and the mutualism dissolving into an antagonistic interaction? For yuccas this may be selective abortion of ovaries that receive numerous yucca moth eggs (Pelmyr and Hurth 1994). For *Epichloë* the mechanism is not known, but a previous study showed larval death rate increased with *Botanophila* egg density on a stroma (Bultman et al. 2000).

During past investigations, researchers observed that male *Botanophila* flies are rare and that a substantial proportion (as much as 30%) of eggs can be non-viable at some sites (Górzyńska et al. 2011; Lembicz et al. 2013). This could indicate the presence of a sexual parasite in fly populations. As with fruit abortion in yucca, a sexual parasite could promote stability of the interacting mutualists by limiting exploitation of *Epichloë* by *Botanophila*. One common type of sexual parasite in insects is *Wolbachia*, a genus of rickettsiae bacteria that lives within the reproductive tissues of its hosts (Werren 1997). In general, these bacteria are thought to be reproductive parasites that may cause a variety of phenotypic changes in their hosts including cytoplasmic incompatibility, parthenogenesis induction, feminisation, and male-killing (Werren et al. 2008).

*Wolbachia* occurs in a vast number of arthropod species as well as filarial nematodes and may be one of the most abundant intracellular genera of bacteria known (Fialho and Stevens 2000; Cordaux et al. 2001; Weeks and Breeuwer 2001; Goodacre et al. 2006), yet it has not been documented in *Botanophila* flies. If a cytoplasmic incompatibility or male-killing strain of *Wolbachia* were to occur in *Botanophila*, it could have important implications for the interaction between the flies and *Epichloë*, such as reducing larval feeding on the fungus. The purpose of our study was to screen *Botanophila* flies for *Wolbachia* infection.

## Materials and methods

### Sample collection and DNA extraction

We collected *Epichloë*-infected grass stems containing stroma with *Botanophila* brood chambers from sites in both Europe and the USA in May and June and stored them in 80% ethanol for transportation. The larvae were removed by cutting open the brood chambers and using forceps to transfer larvae to vials of 80% ethanol for storage until DNA extraction could be performed. DNA extraction was performed on single larvae using the DNeasy Blood and Tissue Kit (Qiagen Inc., Valencia, CA, USA) according to manufacturer’s instructions, but using a final elution volume of 50 µL.

### Identifying Botanophila species

To determine the species of *Botanophila* larvae we amplified the mitochondrial cytochrome oxidase II (COII) gene from total larval DNA using the modified primer TL2-J-3037 (5ʹ-TAATATGGCAGATTAGTGCA-3ʹ) (Leuchtmann 2007) and primer TD-N-3885 (5′-TTTAGTTTGACATACTAATGTTAT-3′) (Simon et al. 1994; Leuchtmann 2007). Polymerase chain reactions (PCR) were performed in 25 µl volumes containing 8 µl *Taq* PCR Master Mix (Qiagen Inc., Valencia, CA, USA). Amplification was conducted in an Eppendorf Pro thermocycler using a program with the following parameters: 7 min at 94°C; 45 s at 94°C, 45 s at 46°C, and 2 min at 70°C, repeated 30 times; 5 min at 70°C; hold at 4°C. Amplified products were separated using gel electrophoresis in 1.5% agarose gels and visualised by SYBR®Green I (Molecular Probes, Eugene, OR, USA) under UV light to check for proper amplification. PCR amplicons were then purified with Wizard® SV Gel and PCR Clean-Up System (Promega Corp., Madison, WI, USA) according to manufacturer’s instructions. Sequencing reactions were performed in 10 µL volumes using a BigDye Terminator Cycle Sequencing kit (PE Applied Biosystems) with recommended PCR conditions. Both strands of the product were sequenced and were separated on a capillary 3130 Genetic Analyzer (Applied Biosystems). Sequences were identified to species using a nucleotide BLAST (Altschul et al. 1990) to compare them to reference sequences of *Botanophila* distinguished by Leuchtmann (2007) and Leuchtmann and Michelsen (2016). Negative controls without DNA were run with each test to ensure the absence of contamination in reagents.

### Assessing Wolbachia infection

We analysed total DNA extracted from larvae for the presence of *Wolbachia* by using PCR to amplify the *Wolbachia* surface protein gene (*wsp*) with primers wsp-F1 (GTCCAATARSTGATGARGAAAC) and wsp-R1 (CYGCACCAAYAGYRCTRTAAA) (Baldo et al. 2006). Reactions were performed in 10 µl volumes containing 5 µl Taq PCR Master Mix (Qiagen Inc., Valencia, CA, USA). Amplification was conducted in an Eppendorf Pro thermocycler using a program with the following parameters: 2 min at 95°C; 30 s at 95°C, 45 s at 53°C, and 1 min at 72°C, repeated 35 times; 6 min at 72°C; hold at 4°C. Amplicons were separated using gel electrophoresis in a 1.5% agarose gel and visualised by SYBR®Green I (Molecular Probes, Eugene, OR, USA) under UV light to determine presence or absence of *Wolbachia*. A positive test for *Wolbachia* resulted in the presence of one band at 603 bp. DNA extracted from an infected specimen of *Mermessus fradeorum* (Araneae) was used as a known positive control for *Wolbachia* (Curry et al. 2015). Negative controls without DNA were run with each test to ensure the absence of contamination in reagents.

## Results

We found *Botanophila* larvae on *Epichloë typhina* infecting *Puccinella distans, Brachypodium pinnatum, Holcus mollis, Holcus lanatus*, and *Dactylis glomerata; Epichloë elymi* infecting *Elymus canadensis* and *Elymus virginicus*; and *Epichloë bromicola* infecting *Bromus benekeni* and *Elymus repens*. We successfully sequenced and identified COII amplicons from 83 fly larvae. In total, we found seven different *Botanophila* species represented (Table 1). Representative sample sequences were submitted to the NCBI GenBank database and can be found under accession numbers MF495863 through MF495888 (NCBI Resource Coordinators 2013). By far the most common species was *Botanophila dissecta*, comprising 50.6% of all larvae collected (Table 1). Two unidentified fly species (*B. sp.5* and *B. sp. 6)* were only found in the USA samples. Five fly species (*B. dissecta, B. lobata, B. phrenione, B. cuspidata*, and *B. laterella*) were found in European samples. *Botanophila lobata* was the only species found in both USA and Europe (Table 2). *Botanophila cuspidata* was collected from *Epichloë typhina* infecting *Puccinella distans* (Table 1), a new record of grass/fungus host for that fly species.

**Table 1.** The number of each fly species found in our total sample and their rates of infection by *Wolbachia*.

Fly species	*N*	# Infected	Infected (%)
*B. cuspidata*	1	1	100
*B. dissecta*	42	35	83.3
*B. laterella*	13	6	46.2
*B. lobata*	12	3	25.0
*B. phrenione*	6	5	83.3
*B. sp 5*	4	3	75.0
*B. sp. 6*	5	5	100
Totals	83	58	69.9

*N* = sample size of fly larvae.

**Table 2.** The *Wolbachia* infection rates of *Botanophila* flies separated by location, fungus and plant species, and fly species.

Location	GPS	Fungus	Plant	Fly Species	*N*	# Infected	Infected (%)
Poland	52° 47.397ʹ N 18° 06.064ʹ E	*E. typhina*	*P. distans*	*B. dissecta*	5	2	40.0
				*B. laterella*	1	1	100.0
				*B. cuspidata*	1	1	100.0
		*E. bromicola*	*E. repens*	*B. lobata*	4	0	0
	52° 46.544ʹ N 18° 06.190ʹ E	*E. typhina*	*P. distans*	*B. phrenione*	3	3	100.0
				*B. laterella*	1	1	100.0
	51° 59.29ʹ ***N*** 17^o^ 9.262ʹ W	*E. typhina*	*B. pinnatum*	*B. dissecta*	6	3	50.0
	51^o^ 54.394ʹ N 17^o^ 2. 587ʹ W	*E. bromicola*	*B. benekeni*	*B. lobata*	1	1	100.0
				*B. laterella*	1	0	0
	52^o^ 46.083ʹ N 17^o^ 92.444ʹ W	*E. typhina*	*H. mollis*	*B. dissecta*	3	2	66.7
				*B. lobata*	1	1	100.0
				*B. laterlla*	1	1	100.0
	52° 27.857ʹ N 16° 55.868ʹ E	*E. typhina*	*D. glomerata*	*B. dissecta*	6	6	100.0
			*H. lanatus*	*B. dissecta*	8	8	100.0
			*D. glomerata*	*B. phrenione*	2	2	100.0
			*D. glomerata*	*B. laterella*	1	1	100.0
			*H. lanatus*	*B. laterella*	2	2	100.0
			*H. lanatus*	*B. phrenione*	1	0	0
	52^o^ 15. 277ʹ N 16^o^ 47.577ʹ W	*E. typhina*	*P. nemoralis*	*B. dissecta*	14	14	100.0
Switzerland	47^o^ 20.566ʹ N 8^o^ 37.432ʹ E	*E. typhina*	*B. pinnatum*	*B. laterella*	6	0	0
Oregon (USA)	44° 59.053ʹ N 122° 56.648ʹ W	*E. typhina*	*D. glomerata*	*B. lobata*	2	0	0
Missouri (USA)	40° 14.100ʹ N 92° 41.042ʹ W	*E. elymi*	*E. virginicus*	*B. sp. 5*	3	3	100.0
				*B. lobata*	4	1	25.0
				*B. sp. 6*	5	5	100.0
Oklahoma (USA)	36° 7.053ʹ N 97° 6.298ʹ W	*E. elymi*	*E. canadensis*	*B. sp. 5*	1	0	0
**TOTAL**					**83**	**58**	**69.9**

Of the 83 samples, 58 were positive for the presence of *Wolbachia* (Table 1). The sequence from the *wsp* gene from *Botanophila lobata* can be found under accession number KR109249 (NCBI Resource Coordinators 2013). The incidence of *Wolbachia* infection varied among fly species, with five species (*B. cuspidata, B. dissecta, B. phrenione, B. sp.5*, and *B. sp. 6*) showing high (>75%) infection, one (*B. laterella*) showing intermediate (46.2%) infection, and one (*B. lobata*) showing low (25.0%) infection (Table 1). Comparing infection rates across continents, we found flies from Europe had higher infection (79.0% – 49/62) than those from the US (60.0% – 9/15) (Table 2). Infection rate also varied across fungal host species; larvae collected from *E. typhina* and *E. elymi* had the highest incidence of infection (75.0% – 48/64; 69.2% – 9/13, respectively), while infection of those from *E. bromicola* was much lower (16.7% – 1/6).

## Discussion

The reproductive fitness of sexually reproducing *Epichloë* can depend upon the service of spermatia-vectoring *Botanophila* flies (Bultman et al. 1995). Here we show that *Wolbachia* bacteria are indeed present in the *Botanophila* genus, a relationship not previously recorded. This discovery may help explain the high levels of non-viable *Botanophila* eggs found in the field (Górzyńska et al. 2011; Lembicz et al. 2013) since *Wolbachia* can result in death or improper development of embryos. To confirm this effect of the parasite, further experimentation is required in which adults are cured of the bacterium, allowed to mate, and their progeny assessed.

Beyond simply confirming the presence of *Wolbachia* bacteria, our results give rise to important questions regarding the effect of the bacteria on *Botanophila* and potentially on the flies’ fungal hosts. First, why do we see variation in infection rates among different *Botanophila* species? Our sample sizes are low and it may be that infection rates actually do not differ, so more sampling is warranted. If, however, infection rates do differ among *Botanophila* species, as our data suggest, this could arise due to differences in geographic location, fly species/population, or fungal species (Table 2). Second, how might the fungal species affect *Wolbachia* infection rates of flies? Some *Epichloë* species (i.e. *E. typhina* and *E. elymi*) were visited more frequently by *Wolbachia*-infected flies than were others (i.e. *E. bromicola*). It is unclear if or how fungi might promote or prevent *Wolbachia* infection, yet if possible, such a mechanism could strongly impact fungal fitness by modifying the amount of larval feeding on perithecia and the ascospores they contain. A possible mechanism by which *Epichloë* might alter the infection status of *Botanophila* is through production of antimicrobial agents that could disinfect *Botanophila* of *Wolbachia*. Interestingly, *Epichloë* are known to produce secondary compounds with antimicrobial properties and their quantities can vary dramatically among *Epichloë* species (Koshina et al. 1989; Steinebrunner et al. 2008b). If *Wolbachia*-infected flies are responsible for laying the nonviable eggs we see in the field, the fungus would benefit by receiving the service of cross fertilisation (through flies vectoring spores) while at the same time avoiding destruction of its progeny (as nonviable eggs would not produce larvae). Such a pathway of interaction could help prevent over-exploitation of *Epichloë* by *Botanophila* and could thus lead to stability in this intriguing interaction.
